# Can the Fatigue Severity Scale 7-item version be used across different patient populations as a generic fatigue measure - a comparative study using a Rasch model approach

**DOI:** 10.1186/1477-7525-12-24

**Published:** 2014-02-22

**Authors:** Sverker Johansson, Anders Kottorp, Kathryn A Lee, Caryl L Gay, Anners Lerdal

**Affiliations:** 1 Department of Neurobiology, Care sciences and Society, Karolinska Institutet, Stockholm, Sweden; 2 Department of Family Health Care Nursing, University of California, San Francisco, CA, USA; 3 School of Nursing, University of California, San Francisco, CA, USA; 4 Senior Researcher, Lovisenberg Diakonale Hospital, Oslo, Norway; 5 Researcher, Lovisenberg Diakonale University College, Oslo, Norway; 6 Department of Nursing Science, Institute of Health and Society, Faculty of Medicine, University of Oslo, Oslo, Norway; 7 Division of Neurology, R54, Karolinska University Hospital, Huddinge, Stockholm 141 86, Sweden

**Keywords:** Acquired immunodeficiency syndrome, Fatigue, HIV, Multiple sclerosis, Rasch analysis, Questionnaire, Stroke

## Abstract

**Background:**

Fatigue is a disabling symptom associated with reduced quality of life in various populations living with chronic illnesses. The transfer of knowledge about fatigue from one group to another is crucial in both research and healthcare. Outcomes should be validly and reliably comparable between groups and should not be unduly influenced by diagnostic variations. The present study evaluates whether the Fatigue Severity Scale 7-item version (FSS-7) demonstrates similar item hierarchy across people with multiple sclerosis, stroke or HIV/AIDS to ensure valid comparisons between groups, and provide further evidence of internal scale validity.

**Methods:**

A secondary comparative analysis was performed using data from three different studies of three different chronic illnesses: multiple sclerosis, stroke and HIV/AIDS. Each of these studies had previously concluded that the FSS-7 has better psychometric properties than the original FSS for measuring fatigue interference. Data from 224 people with multiple sclerosis, 104 people with stroke and 316 people with HIV/AIDS were examined. Item response theory and a Rasch model were chosen to analyze the similarity of the FSS-7 item hierarchy across the three diagnostic groups

**Results:**

Cross-sample differences were found for items #3, #5, #6 and #9 for two of the three samples, which raise questions about item validity across groups. However, disease-specific and disease-generic Rasch measures were similar across samples, indicating that individual fatigue interference measures in these three chronic illnesses might still be reliably comparable using the FSS-7.

**Conclusions:**

Some items performed differently between the three samples but did not bias person measures, thereby indicating that fatigue interference in these illnesses might still be reliably compared using FSS-7 scores. However, caution is warranted when comparing fatigue raw sum scores directly across diagnostic groups using the FSS-7. Further studies of the scale are needed in other types of chronic illnesses.

## Background

Fatigue is a common and disabling symptom in many chronic diseases. It often co-varies with depressive symptoms [1-4] and sleep impairment [5,6] and is associated with poorer self-reported health status [7,8] and reduced quality of life [9-12]. Because fatigue is a perceived phenomenon, researchers and clinicians rely on subjective measures to indicate need for intervention or effectiveness of treatment. When 18 fatigue measures used in chronic illness research were reviewed, the Fatigue Severity Scale (FSS) [13] was rated highest on robust psychometric properties [14]. An advantage of the FSS is that it is a short 9-item measure with items formulated as statements about the fatigue experience itself (item #3), what causes fatigue (item #2), and how fatigue interferes with daily life (7 items).

In our prior work [15-17] we used Rasch analysis to evaluate the psychometric properties of the original 9-item version of the FSS within several chronic illness groups, including multiple sclerosis (MS), stroke and HIV/AIDS. These three illnesses were selected because fatigue is a well-documented and prevalent symptom in each of these patients groups [18-25]. The prior studies [15-17] each concluded that a 7-item version of the FSS (FSS-7) has better psychometric properties and is a reliable and valid measure of fatigue *interference* rather than *severity* as indicated by the title of the measure. These studies, as well as a study by Mills et al. [26], provided consistent evidence regarding the relationship between the included items and the underlying latent trait in diagnosis-specific samples. However, we do not know whether the FSS items function similarly across the samples and are thus appropriate for use in comparative studies.

Rasch analysis is a useful tool for evaluating these types of psychometric properties. It is part of the group of modern psychometric approaches in item response theory used to evaluate the relative endorsement, or hierarchy of items within a measure. Rasch models support the process of validation analysis by providing a transformation of an ordinal score into a linear, interval-level variable. The Rasch model shows what item responses would be expected if interval scale measurement is to be achieved. Actual response patterns, identified in a questionnaire with a set of items intended to be summed together, are tested against what would be expected by the model. The hierarchical ordering of items within the scale can also be affirmed [27]. That is, the items can be arranged in order of relative difficulty based on their likelihood of being endorsed given an underlying level of the measured construct.

Although the original FSS has been used to measure fatigue in a variety of different populations, we found no studies that explored the stability of the item hierarchy across different disease populations. In this context, “stability” does not mean reliability as defined in classical test theory, but rather reflects the similarity, consistency or invariability of the item hierarchies across groups. If item hierarchies vary across groups (some items are easier or harder to agree with for one group compared to another group), the resulting scores will be biased. Rasch analysis addresses and evaluates the impact of such issues using differential item functioning (DIF) [27]. Studies encompassing several disease groups are critical for integrating evidence to transfer knowledge about potential interventions from one clinical specialty to another. Measures such as the FSS must therefore be evaluated across diagnostic groups to ensure that the scale functions comparably across groups and is not overly influenced by diagnostic variations.

Thus, the purpose of this study was to evaluate whether the FSS-7 demonstrates a similar item hierarchy across three chronic illness groups (MS, stroke and HIV/AIDS) to ensure valid comparisons between groups and provide further evidence of internal scale validity. We conducted a secondary analysis of FSS data from three different studies of samples with potential diagnostic differences in item hierarchy. Each of these studies had previously concluded that the FSS-7 has better psychometric properties for measuring fatigue interference than the original FSS-9 [15-17], and this study aims to determine whether the FSS-7 can be validly and reliably compared across illness groups.

## Methods

### Samples and procedures

#### MS

Data were collected in a longitudinal study of functioning and disability in people with MS at an outpatient MS specialist clinic in Stockholm, Sweden. All people over 18 years of age with definite MS according to the Poser criteria [28] who were scheduled for an outpatient appointment with their neurologist were eligible. Data were collected at baseline and at 6-month intervals for 2 years from 2002 to 2004. Results have been reported elsewhere [2,19]. A total of 227 people with MS were enrolled in the study, at baseline they had been diagnosed with MS for a mean of 14 years (standard deviation [SD] 10 years). Data on age and sex were collected from medical records. The Swedish version of the original FSS was completed via face-to-face interviews by 224 respondents at baseline; these data were used in the current analysis.

#### Stroke

Data were collected at 6-month intervals for 2 years from 2007 to 2009 as part of a Norwegian longitudinal study of people who presented with a first-ever clinical stroke according to the ICD–10 [29], were age 18 years or older and had sufficient cognitive function to participate. A detailed description of the study has been previously reported [22]. Data on age and sex were collected from medical records. The Norwegian version of the original FSS was completed via face-to-face interviews or mailed questionnaires. The 12-month data (n = 104) were used for this analysis to better approximate the chronic phase of the illness assessed in the other two samples.

#### HIV/AIDS.

Data were collected from 2005 to 2007 as part of a longitudinal study of people with HIV/AIDS in San Francisco, USA [24]. The study was designed to characterize the symptom experience of people with HIV/AIDS and to identify biological and genetic markers of the symptom experience. Eligible participants were English-speaking, 18 years or older, and diagnosed with HIV/AIDS at least 30 days before enrollment. Individuals were excluded if they currently used illicit drugs, worked nights, had been pregnant in the previous 3 months, or reported having a diagnosed sleep disorder, schizophrenia, bipolar disorder, or dementia. Participants completed a baseline assessment, and those not reporting significant sleep disturbance or fatigue continued with assessments at 6-month intervals for up to 2 years. At baseline they had been diagnosed with HIV for a mean of 12 years (SD 7 years). The results have been reported elsewhere [24]. Self-report questionnaires were used to collect data on age, gender, race/ethnicity, level of education, clinical characteristics, and concurrent symptoms. At baseline the original FSS version was completed by 316 respondents, and these data were used in the current analysis.

### Data collection

Fatigue was measured with the original FSS developed by Krupp [13]. Each item is scored on a 7–point Likert scale ranging from 1 (“strongly disagree”) to 7 (“strongly agree”). The mean score of the 9 items has commonly been used to estimate fatigue interference. Findings from recent studies in people with MS [15,26], stroke [16], and HIV/AIDS [17] show that items #1 (“My motivation is lower when I am fatigued”) and #2 (“Exercise brings on my fatigue”) do not show acceptable goodness-of-fit to the Rasch model and thus should not be included in the mean score of the FSS. As these findings seem to be congruent, we chose in this study to use the remaining seven items (FSS-7) for the analysis of item hierarchy similarity. These items assess demonstrated characteristics of fatigue and focus on the extent to which fatigue interferes with various aspects of daily functioning [17].

### Ethical considerations

The MS study was approved by the ethics committee of Karolinska Institutet in Stockholm, Sweden (reference No 449/01). The stroke study was approved by the Regional Medical Research Ethics Committee of Health East of Norway and the Norwegian Data Inspectorate, Norway (reference No 2009/1468 S-07027b). The HIV study was approved by the Committee of Human Research at the University of California, San Francisco, USA (reference No 10–01357).

### Data analysis

Descriptive statistics were used to summarize sample socio-demographic characteristics and FSS-7 scores. Statistically significant differences in characteristics were analyzed using chi-square test for categorical data and independent sample t-test for continuous data. The level of significance was set to *p <* 0.05 and all tests were two-tailed.

Item response theory and a Rasch model were chosen to analyze the similarity of the FSS-7 item hierarchy across the three diagnostic groups. The Rasch model takes each item scored and adjusts the final person measure based on relative differences in item severity. Rasch models are also suitable for handling data where items may be missing, so no participant was excluded due to specific missing values [30-32].

The WINSTEPS analysis software program, version 3.69.1.16 [33] was used to conduct the Rasch analyses in this study. Such an analysis first converts raw item scores from a questionnaire into equal-interval measures using a logarithmic transformation of the odds of the actual responses. These converted values can then also be used to examine whether the scale items measure a one-dimensional construct [30,34]. Based upon the actual pattern of responses, the Rasch transformation simultaneously results in an estimation of a person’s fatigue interference measure as well as measures (calibrations) of each item along a calibrated continuum: from items that are easier to agree with to items that are harder to agree with. Rasch models are probabilistic and based on theoretical assertions against which the actual pattern of responses is validated. Although the FSS-7 uses a generic rating scale from 1 to 7, it may not function in a similar manner across all items, as items may demonstrate different response patterns even though the same generic rating scale is used. Therefore, a partial credit model, developed for scales where ratings may differ across items, was applied to the FSS-7 in this study.

Initially, all diagnostic subgroups were analyzed together, resulting in disease-generic individual fatigue interference measures for each participant. Analyses of variance (ANOVAs) were then conducted to compare the fatigue interference measures between the three diagnostic groups. If a significant main effect was found, additional Tukey post hoc tests with a significance level set at *p* < 0.05 were then used to identify significant pairwise differences between the diagnostic groups.

Secondly, three separate Rasch analyses were generated in order to explore the diagnosis-specific FSS-7 item hierarchies. A number of DIF analyses were then performed to evaluate the similarity of relative FSS-7 item calibrations across the three diagnostic groups, thereby determining whether bias existed for the items among the groups. The magnitude of DIF was evaluated using the Mantel-Haenszel statistic for polytomous scales using log-odds estimators [35,36] in the WINSTEPS program. Although a Bonferroni correction yielding a 1% alpha is commonly used [27], we also report results with *p <* 0.05 to more conservatively evaluate the likelihood of item bias. For the FSS-7 item hierarchies to be considered similar across diagnostic groups and support further evidence of cross-diagnostic scale validity, we expected that no item should have a significant DIF between pairs of diagnostic groups.

Finally, we used the disease-specific FSS-7 item hierarchies and generated disease-specific measures for each participant as well. We then compared the individual measures generated from each disease-specific FSS-7 scale with the original individual measures from the disease-generic FSS-7 scale. This analysis aimed to explore the impact of different item hierarchies (different tests) on the individual measures using Differential Test Functioning (DTF). We used standardized *z*-comparisons to evaluate whether the measures generated from the disease-specific and disease-generic FSS-7 scales differed significantly from each other, considering the level of precision (standard error) of each individual fatigue interference measure.

## Results

In the present study, data from 224 people with MS, 104 people with stroke, and 316 people with HIV/AIDS were analyzed. Socio-demographic characteristics and FSS-7 mean scores for the three samples, as well as significant differences between the samples, are shown in Table 1.

**Table 1:** Sample socio-demographic characteristics and fatigue severity scale-7 mean scores

**Socio-demographic variables**	**MS N = 224 (Group A)**	**Stroke N = 104 (Group B)**	**HIV/AIDS N = 316 (Group C)**	**Significant differences between groups (*p*** **< 0.05)**
Age: years (M ± SD)*	46.5 ± 12.4	68.0 ± 12.8	45.1 ± 8.4	A,C < B
Range	20 – 75	29 – 91	22 – 77	
Years since diagnosis (M ± SD)*	14 (10)	1 (0.1)	12 (7)	A,C > B
Range	0 – 44	1 – 1	0 – 27	
Gender: n (%)				
Women	149 (68)	41 (39)	77 (24)	A > B > C
Men	(75 (32)	63 (61)	216 (69)	
Transgender			23 (7)^†^	
Living with partner: n (%)	152 (69)	70 (67)	109 (35)	A,C > B
In paid work: n (%)	97 (43)	25 (24)	42 (13)	A > B > C
FSS-7 (M ± SD)*	4.7 ± 1.7	3.9 ± 1.6	3.8 ± 1.7	A > B,C
Range	1.0 – 7.0	1.0 – 7.0	1.0 – 7.0	

*(Mean ± Standard Deviation).

^†^Transgender was not included in the analysis of differences between groups with regard to gender.

### Differences in fatigue between diagnostic groups

The results of the one-way ANOVA for the FSS-7 interference measures revealed a significant main effect for diagnostic group (F [19.81] *p* < 0.001). Tukey post hoc tests indicated that the MS group had significantly higher FSS-7 scores than both the stroke group (*p* < 0.001) and the HIV group (*p* < 0.001), but the stroke group and HIV group did not differ significantly from each other (*p* = 0.79). Fatigue interference (FSS-7) scores were converted into equal-interval measures that can range from 0 to 100, and the means, standard deviations and ranges for the three groups were: 1) MS: 56.1 ± 16.1 (range 6.4 – 91.9); 2) stroke: 48.6 ± 14.6 (range 6.4 – 91.9), and 3) HIV: 47.3 ± 17.3 (range 6.4 – 91.9).

### Stability of relative FSS item calibrations

**Figure 1:**
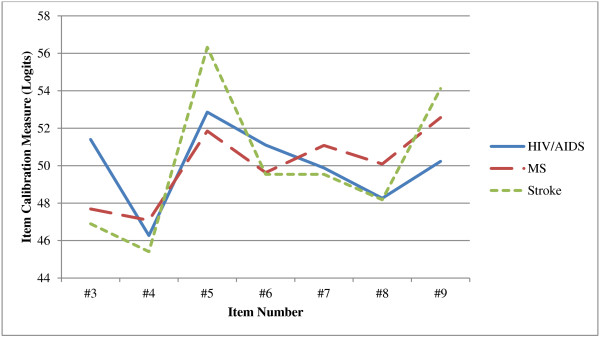
Fatigue Severity Scale-7 item difficulty calibrations for three samples with HIV/AIDS (n = 316), MS (n = 224), and stroke (n = 104).

The relative FSS-7 item calibration hierarchies are presented in Table 2 for people with MS, in Table 3 for people with stroke, and in Table 4 for people with HIV/AIDS. Relative FSS-7 item difficulty calibrations for all three samples are also illustrated in Figure 1.

**Table 2:** Fatigue severity scale-7: item hierarchy demonstrated in people with MS

	Measure (SE*) (logit)	**Mean (SD**^**†**^**) (raw score)**	**Item**
Harder items to agree with	52.57 (.55)	4.37 (2.19)	Item 9: Fatigue interferes with my work, family or social life
	51.85 (.59)	4.43 (2.05)	Item 5: Fatigue causes frequent problems for me
	51.08 (.55)	4.60 (2.23)	Item 7: Fatigue interferes with carrying out certain duties and responsibilities
	50.09 (.55)	4.79 (2.22)	Item 8: Fatigue is among my three most disabling symptoms
	49.63 (.55)	4.83 (2.22)	Item 6: My fatigue prevents sustained physical functioning
	47.69 (.62)	4.83 (1.88)	Item 3: I am easily fatigued
Easier items to agree with	47.08 (.59)	5.06 (2.02)	Item 4: Fatigue interferes with my physical functioning

Items completed by people with MS: all items were completed by n = 224.

*Standard error; ^†^Standard deviation.

**Table 3:** Fatigue severity scale-7: item hierarchy demonstrated in people with stroke

	Measure (SE*) (logit)	**Mean (SD**^**†**^**) (raw score)**	**Item**
Harder items to agree with	56.32 (.96)	3.23 (1.77)	Item 5: Fatigue causes frequent problems for me
	54.12 (.95)	3.46 (1.91)	Item 9: Fatigue interferes with my work, family or social life
	49.54 (.95)	3.93 (2.17)	Item 6: My fatigue prevents sustained physical functioning
	49.54 (.95)	3.95 (1.95)	Item 7: Fatigue interferes with carrying out certain duties and responsibilities
	48.18 (.95)	4.10 (2.24)	Item 8: Fatigue is among my three most disabling symptoms
	46.90 (.96)	4.23 (1.90)	Item 3: I am easily fatigued
Easier items to agree with	45.41 (.97)	4.38 (1.67)	Item 4: Fatigue interferes with my physical functioning

Items completed by people with stroke: items 3, 4, 5, 7, 8 and 9, n = 104; item 6, n = 103.

*Standard error; ^†^Standard deviation.

**Table 4:** Fatigue severity scale-7: item hierarchy demonstrated in people with HIV/AIDS

	Measure (SE*) (logit)	**Mean (SD**^**†**^**) (raw score)**	**Item**
Harder items to agree with	52.86 (.50)	3.41 (2.06)	Item 5: Fatigue causes frequent problems for me
	51.40 (.49)	3.59 (2.01)	Item 3: I am easily fatigued
	51.10 (.49)	3.65 (1.99)	Item 6: My fatigue prevents sustained physical functioning
	50.23 (.49)	3.76 (2.13)	Item 9: Fatigue interferes with my work, family or social life
	49.88 (.49)	3.80 (2.01)	Item 7: Fatigue interferes with carrying out certain duties and responsibilities
	48.26 (.49)	4.00 (2.24)	Item 8: Fatigue is among my three most disabling symptoms
Easier items to agree with	46.27 (.50)	4.28 (2.08)	Item 4: Fatigue interferes with my physical functioning

Items completed by people with HIV/AIDS: item 3, n = 313; item 4, n = 312; item 5, n = 315; items 6, 7 and 8, n = 314; item 9, n = 316.

*Standard error; ^†^Standard deviation.

In Table 5, significant DIF comparisons by diagnostic groups are presented for each of the seven items. Differences for item #3 (“I am easily fatigued”) were found between MS and HIV/AIDS groups (*p* < 0.01) as well as between stroke and HIV/AIDS groups (*p* < 0.01). Furthermore, differences were found for item #5 (“Fatigue causes frequent problems for me”) between MS and stroke groups (*p* < 0.01) and for item #6 (“My fatigue prevents sustained physical functioning”) between MS and HIV/AIDS groups (*p* < 0.05). There were also differences found for item #9 (“Fatigue interferes with my work, family, or social life”) between MS and HIV/AIDS groups (*p* < 0.01) as well as between stroke and HIV/AIDS groups (*p* < 0.05). Our set criterion to support evidence of scale validity was not met, as multiple items demonstrated significant relative DIF when comparing diagnostic groups.

**Table 5:** Differential item functioning of fatigue severity scale-7 by diagnostic groups

**Item**	**Differential item functioning**
3	MS – HIV/AIDS** Stroke – HIV/AIDS**
4	None
5	MS – Stroke**
6	MS – HIV/AIDS*
7	None
8	None
9	MS – HIV/AIDS** Stroke – HIV/AIDS*

*Indicates significant differences (*p < 0.05; **p < 0.01) using Mantel-Haenszel statistic for polytomous scales. The statistic indicated significant differential item function by diagnostic group for items #3 (a relatively more difficult item for the HIV/AIDS group compared to the MS and stroke groups, #5 (a relatively easier item for the MS group compared to the stroke group, #6 (a relatively easier item for the MS group compared to the HIV/AIDS group, and #9 (a relatively easier item for the HIV/AIDS group compared to the MS or stroke groups). Item calibrations for items #4, #7, and #8 did not differ by diagnostic group.

### Differences between the generated disease-specific and disease-generic Rasch person measures of the FSS-7

To compare the generated disease-specific scores from the three groups with the generated disease-generic scores (used in the ANOVA comparison), a standardized *z*-comparison was used. None of the 644 person-measures had a *z*-value exceeding ±1.96 (*z*-values ranged from −0.69 to 0.78). These results suggest that the disease-specific item hierarchies do not heavily influence the individual measures, as results were comparable to the disease-generic measure. This was the case even though the three diagnostic groups differed with respect to their relative FSS-7 item calibrations.

## Discussion

In the present study, fatigue interference, as measured with the FSS-7, was compared in three chronic illness samples (MS, stroke, and HIV/AIDS). Overall, the MS sample demonstrated more fatigue interference than the stroke and HIV samples. However, four of the seven items functioned differently for two of the three samples, thereby failing to meet the set criterion for stability across diagnostic groups and raising questions about item validity across groups. Nonetheless, when disease-specific scores were compared to the disease-generic scores, person measures were generally placed in the same position when considering the level of precision evident in individual standard errors, thus indicating that individual fatigue interference in these three chronic illnesses might still be reliably comparable when using the FSS-7.

The MS group had more fatigue interference than both the older group of people with stroke and the HIV group of similar age. These differences may be due to the fact that almost half of the MS sample was employed and more were partnered compared to the other two groups. These findings are also consistent with prior reports of higher prevalence rates of fatigue among people with MS (55% to 83%) [18-20] than in other groups living with chronic illness, including stroke (24% to 77%) [21,22] and HIV (37% to 65%) [23-25].

With respect to the specific FSS-7 items, people with HIV/AIDS were less easily fatigued (item #3) than people with MS or stroke. Given the higher rates of employment among those with MS and stroke, these groups may be more likely than people with HIV/AIDS to encounter situations that demand energy, and thus contribute to fatigue. In addition, living with a partner, which was more common among the people with MS or stroke in this study, has been associated with increased fatigue [2]. Living with a partner might interfere with adjustments to one’s level of activity in an attempt to manage fatigue and may help explain the group differences on item #3. In fact, such contextual issues may potentially be influencing the hierarchies more than the specific chronic illness.

People with MS were more likely than people with stroke to agree that fatigue causes frequent problems for them (item #5). However, differences in the specific position of each item may not be as important as overall hierarchical order, and while item #5 was the hardest item to agree with for people with stroke and for people with HIV/AIDS, it was still the second hardest item to agree with for people with MS. The fact that item #5 was relatively hard for all three diagnostic groups to agree with may also reflect how people with fatigue learn to cope, and thereby reduce its impact on their lives. People who experience low energy often reduce their activity according to their perceived capacity for mental and physical work, and thus their fatigue may not necessarily result in frequent problems [37]. In addition, the finding that fatigue interference more easily causes problems for people with MS could reflect ongoing disease activity or the progression of impairment in people with MS, which might make it more difficult for them to compensate for fatigue-related problems.

The samples with MS and HIV/AIDS differed significantly regarding the effect of fatigue on sustained physical functioning (item #6), a difference which might be explained by the fact that people with MS are more likely to be bothered by heat sensitivity [3], which is often regarded as a barrier to being physically active. In addition, people with HIV/AIDS were more likely than people with MS or stroke to report that fatigue interferes with their work, family, or social life (item #9). This finding may be associated with the experience of stigma, isolation, and medical disability documented in people with HIV/AIDS. The extent to which fatigue interference adds to the challenges faced by people with HIV/AIDS needs further research to better understand how it affects one’s relationships and work life.

The three samples in this study not only differed in diagnosis, but also differed with respect to nationality, culture, and language. Thus, the potential influence of the different social systems in Sweden, Norway, and the USA on whether and how people perceive fatigue as problematic needs to be considered, particularly since various social and health care systems place different demands on individuals living with chronic illness. These additional differences across samples might be considered a limitation, however, the cultural and language differences would be expected to increase differences across diagnostic groups, and yet in our prior psychometric studies of these three groups [15-17], the results have been quite congruent, providing further evidence that the FSS-7 is functioning well in different cultural and diagnostic populations.

The DIF findings pose an interesting interpretative challenge from a test validity perspective. In general, in order to conclude that a test is not biased and provide evidence of cross-diagnostic scale validity, we would expect there to be no DIF related to diagnosis. Our findings did not support the validity of FSS-7 from this perspective. However, the broader issue here relates to whether the findings of systematic bias are the result of true clinical differences between the diagnostic groups. The above discussion about the specific DIF findings suggests that these differences may have logical and empirical support, and therefore not be a major threat to internal scale validity. Still, it is important to evaluate whether such item calibration differences will have an impact when comparing measures between diagnostic groups. The disease-specific Rasch person measures of the FSS-7 did not differ from the disease-generic Rasch person measures, and thus people are placed in relatively the same place along a continuum of fatigue interference, irrespective of whether the generated Rasch measures are disease-specific or disease-generic. Based on the findings of this study, we can therefore conclude that a generic tool of fatigue may generate unique diagnostic profiles for different target groups, and at the same time still generate individual measures of fatigue that are not overly biased by diagnosis.

People with MS, stroke, or HIV/AIDS do experience fatigue interference differently, as evidenced by the varying relative item hierarchies and the diagnostic differences in four of the FSS-7 items. Therefore, comparisons of FSS-7 scores in these populations should be performed with these issues in mind. The empirical findings from this study demonstrate that there is a parallel need to also evaluate DTF when exploring DIF. There is a balancing act between having sensitive clinical tests that identify unique clinical profiles for diagnostic groups, and at the same time allow for valid comparisons of the generated measures across different samples.

The findings of this study also raise important issues about the use of generic or specific outcome measures in health care research. Many concepts used in research are generic in nature (e.g., quality of life, fatigue, ADL ability) and not specifically constructed with a particular diagnosis in mind. By using generic measures and allowing comparisons between diagnostic groups, we can generate new knowledge that can be used across diagnoses to generate a deeper understanding of a target phenomenon such as “fatigue”, as well as evaluate potential therapeutics. However, today the trend is toward using diagnosis-specific outcome measures for generic phenomenon. Although we might assume that this development allows the test developers to generate more specific items that may match the unique profile of that specific population, there are marked similarities across these diagnosis-specific measures.

The aim of this study was to evaluate whether the FSS-7 demonstrated similar item hierarchies across people with MS, stroke, and HIV/AIDS. Given that four items did not perform in a similar hierarchy for the clinical groups, caution is warranted when comparing fatigue across diagnostic groups using the FSS-7. However, a replication of this study using different diagnostic groups is needed to validate our results on a group level. This could be performed with larger data sets and/or with item split techniques [38]. Such studies on item hierarchies in different groups may also provide a better understanding of diagnostic profiles – if and how different groups experience a specific phenomenon. Such understanding can also be of particular importance for the evaluation of the effectiveness of targeted interventions for different diagnostic groups.

Results from the present study are also important to consider for clinical trials. The effects of a fatigue intervention should not only provide results on sum scores but should also report changes in the specific item hierarchies, particularly since an item-specific change might be the reason an intervention had the reported effect in a specific diagnostic group or failed to demonstrate the desired effect. In addition, according to the findings of this study, both an item reduction and Rasch analysis of the raw scores of the original FSS would be required to facilitate transfer of such knowledge across diagnostic groups. Earlier studies showed non-linear relationships between the item raw sum scores and Rasch-generated measures of the original FSS, which indicates the risk of either over- or under-estimating fatigue interference in people with chronic illness by continuing to use the item raw sum score of the original FSS [15]. An additional benefit of using Rasch-generated measures is the provision of an individual precision estimate (i.e., standard error) for each client, which minimizes the risk of overestimating changes or differences on an individual level, as demonstrated in this study when exploring DTF.

### Limitations

In addition to the cultural and language differences mentioned above, the main limitation of this study is that the three diagnostic groups were not matched on any potentially confounding variables which might also explain the observed group differences. There were limited clinical and socio-demographic data available for these samples, and additional detail regarding potentially confounding variables may have facilitated interpretation of the results, particularly regarding DIF. One possible strategy could have been to match the groups regarding age and gender. However, as these socio-demographic differences probably reflect true differences between these populations, this strategy might result in findings that have higher internal validity, but limited external validity. In addition, possible variations in depressive symptoms in the different groups were not taken into account as part of this study. The severity of depressive symptoms can influence how fatigue is experienced [39] and thus, might have influenced the results. Another possible confounder which was not taken into account in the analyses is the fatigue-associated side effects of various medications. Fatigue is a common side effect of immunomodulatory medications commonly used to treat both MS [40] and HIV [41], and future studies should consider the potential influence of these and other medications. Although the variation in sample size between the three groups may have some impact upon their representativeness and generalizability, a sample size of at least 100 will generate relatively stable item estimates. Thus, the variation in sample sizes between the groups is likely to have minimal impact on the results. Finally, the current findings can only be generalized to the chronic illnesses included in this study and warrant exploration in other populations.

## Conclusions

When fatigue interference, as measured with the FSS-7, was compared across three chronic illnesses (MS, stroke, and HIV/AIDS), four items functioned differently between the samples. However, when comparing the disease-specific scores of the three samples with the disease-generic scores, person measures were placed in relatively the same manner, thereby suggesting that fatigue interference in these chronic illnesses might still be reliably compared using FSS-7 scores. Nonetheless, caution is warranted when comparing FSS-7 measures across diagnostic groups, and further studies of the FSS-7 and other symptom measures are needed.

## Abbreviations

MS: Multiple sclerosis; HIV: Human immunodeficiency virus; AIDS: Acquired immunodeficiency syndrome; FSS: Fatigue severity scale, original version; FSS-7: Fatigue severity scale, 7-item version; ICD-10: International statistical classification of diseases and related health problems - Tenth revision; DIF: Differential item functioning; ANOVA: Analysis of variance; DTF: Differential test functioning.

## Competing interests

The authors declare that they have no competing interests.

## Authors’ contributions

SJ collected the data in the MS study, participated in the analyses of data and drafted the manuscript. AK analyzed and interpreted the data and contributed to the writing of the manuscript. KAL and CLG participated in the collection of data in the HIV/AIDS study, participated in the analyses of data and contributed to the writing of the manuscript. AL participated in the collection of data in the stroke study, participated in the analyses of data and contributed to the writing of the manuscript. All authors read and approved the final manuscript.
